# An Agonist of the CXCR4 Receptor Strongly Promotes Regeneration of Degenerated Motor Axon Terminals

**DOI:** 10.3390/cells8101183

**Published:** 2019-09-30

**Authors:** Samuele Negro, Giulia Zanetti, Andrea Mattarei, Alice Valentini, Aram Megighian, Giulia Tombesi, Alessandro Zugno, Valentina Dianin, Marco Pirazzini, Silvia Fillo, Florigio Lista, Michela Rigoni, Cesare Montecucco

**Affiliations:** 1 Department of Biomedical Sciences, University of Padua, 35131 Padua, Italy; samuele.negro1987@gmail.com (S.N.); gzanetti89@gmail.com (G.Z.); aram.megighian@gmail.com (A.M.); marcopiraz@gmail.com (M.P.); 2 Department of Pharmaceutical and Pharmacological Sciences, University of Padua, 35131 Padua, Italy; andrea.mattarei@unipd.it (A.M.); ali.valentini23@gmail.com (A.V.); alessandro.zugno@phd.unipd.it (A.Z.); valedianin@gmail.com (V.D.); 3 Padua Neuroscience Institute, 35131 Padua, Italy; 4 Department of Biology, University of Padua, 35131 Padua, Italy; giulia.tombesi@phd.unipd.it; 5 Center of Medical and Veterinary Research of the Ministry of Defence, 00184 Rome, Italy; silviafillo@gmail.com (S.F.); romano.lista@gmail.com (F.L.); 6 CNR Institute of Neuroscience, 35131 Padua, Italy

**Keywords:** CXCR4 receptor, neurodegeneration, neuroregeneration, neuromuscular junction, motor neuron

## Abstract

The activation of the G-protein coupled receptor CXCR4 by its ligand CXCL12α is involved in a large variety of physiological and pathological processes, including the growth of B cells precursors and of motor axons, autoimmune diseases, stem cell migration, inflammation, and several neurodegenerative conditions. Recently, we demonstrated that CXCL12α potently stimulates the functional recovery of damaged neuromuscular junctions via interaction with CXCR4. This result prompted us to test the neuroregeneration activity of small molecules acting as CXCR4 agonists, endowed with better pharmacokinetics with respect to the natural ligand. We focused on NUCC-390, recently shown to activate CXCR4 in a cellular system. We designed a novel and convenient chemical synthesis of NUCC-390, which is reported here. NUCC-390 was tested for its capability to induce the regeneration of motor axon terminals completely degenerated by the presynaptic neurotoxin α-Latrotoxin. NUCC-390 was found to strongly promote the functional recovery of the neuromuscular junction, as assayed by electrophysiology and imaging. This action is CXCR4 dependent, as it is completely prevented by AMD3100, a well-characterized CXCR4 antagonist. These data make NUCC-390 a strong candidate to be tested in human therapy to promote nerve recovery of function after different forms of neurodegeneration.

## 1. Introduction

A variety of damages, such as mechanical traumas and autoimmune or neurotoxic attacks, can affect peripheral neurons, owing to their anatomical exposure. Together with its essential physiological role, this explains why the peripheral nervous system (PNS) has retained the capability to regenerate its neurons through evolution, at variance from the central one [1,2,3,4,5]. Understanding the mechanisms of and players in the regeneration process is of crucial importance, as it will lead to the discovery of novel drugs to support current protocols of rehabilitation after accidents or other damage to PNS neurons. It is also likely that these drugs may exert similar properties toward injured central neurons.

G protein-coupled receptors are a large protein family that detects molecules outside the cell and activates internal signal transduction pathways and cellular responses through G protein-coupled receptor kinases [6,7]. We recently reported that a signaling axis composed of a member of this family, CXCR4, and its natural ligand, CXCL12α, contributes to the functional and anatomical recovery of the neuromuscular junction (NMJ) following an acute nerve terminal damage [8]. In response to a motor axon terminal injury perisynaptic Schwann cells (PSCs) synthetize and release the chemokine CXCL12α, which interacts with its G protein-coupled receptor CXCR4 expressed on the tip of the re-growing axon, thus promoting muscle re-innervation. Interfering with either the two members of the axis delays regeneration, while exogenous administration of recombinant CXCL12α accelerates the process [8], thus advocating the CXCR4 receptor as a pharmacological target and the CXCL12α ligand as a drug promoting nerve regeneration.

CXCL12α was discovered by screening for proteins carrying a signal sequence either for secretion or for incorporation into the plasma membrane [9], and by a search for growth factors for pre-immune cells [10]. Indeed CXCL12α, also termed stromal cell-derived factor 1 (SDF-1), is a growth factor for bone marrow pre-B cells [10], and it plays a variety of additional functions in the immune system. Later on, CXCL12α was shown to be involved in the development of various regions of the central nervous system (CNS) [11,12,13,14,15,16,17,18,19,20,21], and its receptor appears to be implicated in several neurodegenerative diseases [22].

CXCR4 is also a co-receptor for HIV entry into human cells [23]. Consequently, it is not surprising that an intensive search for pharmacological modulators of CXCR4, either antagonists or agonists, displaying more convenient pharmacological properties than CXCL12α is ongoing [24,25,26,27,28,29,30,31,32,33,34,35,36,37,38,39]. Using a remarkable structural and computational chemistry approach, a series of ligands of the CXCR4 receptor, consisting of small non-peptide organic molecules, was recently identified [40]. A screening of the properties of these molecules identified five novel agonists. One of them, dubbed NUCC-390, displayed the highest capability to activate the CXCR4 receptor in a battery of specific in vitro assays [40].

As the properties of NUCC-390 were investigated [40], but the details of its chemical synthesis were not given, we designed a very convenient synthesis of NUCC-390, which is described here. The molecule was tested for its ability to promote the regeneration of motor axon terminals in a mouse model of reversible degeneration induced by the black widow spider neurotoxin α-Latrotoxin (α-LTx). We found that NUCC-390 recapitulates the activity of CXCL12α, shortening the time-course of neurotransmission rescue at the NMJ. These findings propose NUCC-390 as a candidate molecule to be tested in humans to support functional recovery from peripheral nerve damages and, possibly, for CNS neurons as well.

## 2. Materials and Methods

### 2.1. Reagents

*NUCC-390 synthesis: N*-4-(Boc-amino)cyclohexanone (750816), diethyl oxalate (75712), lithium bis(trimethylsilyl)amide (225770), trifluoroacetic acid (302031), potassium carbonate (347825), 4-vinylpyridine (V3204), 1-[Bis(dimethylamino)methylene]-1H-1,2,3-triazolo [4–5-b]pyridinium 3-oxid hexafluorophosphate (HATU) (445460), piperidine (104094), potassium hydroxide (484016), *N,N*-diisopropylethylamine (D125806), solvents were from Sigma–Aldrich (Milan, Italy) and were used as received. Propylhydrazine dihydrochloride was prepared according to adapted literature procedures [41].

Cytosine β-d-arabinofuranoside hydrochloride (C6645), DNase I from bovine pancreas (DN25), poly-l-lysine hydrobromide (P1274), laminin (L2020) and trypsin (T4799) were from Sigma–Aldrich (Milan, Italy). µ-Conotoxin GIIIB and α-LTx were purchased from Alomone (Jerusalem, Israel). AMD3100 was from Abcam (120718, Cambridge, UK). Primary antibodies: anti-β_3_-tubulin (302 302) from Synaptic System (Goettingen, Germany), anti-syntaxin-1A/1B was produced in rabbit in our laboratory and previously characterized [42]. Secondary antibodies Alexa Fluor 488, Alexa Fluor 555 conjugated, and α-BTx-Alexa-555 were from Thermo Scientific (MA, USA).

### 2.2. NUCC-390 Synthesis

NUCC-390 organic synthesis is described in detail in the Supplementary Material, while here, we report the instrumental operations employed. Briefly, TLC (thin-layer chromatography) plates were run on silica gel supported on plastic (Macherey–Nagel Polygram^®^SIL G/UV254, silica thickness 0.2 mm, Duren, Germany) and visualized by UV detection and/or permanganate stain. Flash chromatography was performed on silica gel (Macherey–Nagel 60, 230–400 mesh granulometry (0.063–0.040 mm) under air pressure. NMR spectra were recorded with a Bruker AMX300 spectrometer operating at 300 MHz for ^1^H-NMR and 75 MHz for ^13^C-NMR and a Bruker Avance S spectrometer (Milan, Italy) operating at 400 MHz for ^1^H-NMR and 101 MHz for ^13^C-NMR. Chemical shifts (δ) are given in ppm relative to the signal of the solvent. HPLC/ESI-MS analyses and mass spectra were performed with an 1100 Series Agilent Technologies (Milan, Italy) system, equipped with binary pump (G1312A) and MSD SL Trap mass spectrometer (G2445D SL) with ESI source. ESI-MS positive spectra of reaction intermediates and the final purified product were obtained from solutions in acetonitrile, eluting with a water:acetonitrile = 1:9 mixture containing 0.1% formic acid. The purity of the final product was >95%, as assessed by HPLC-UV analysis.

### 2.3. Animals

All animals were handled by specialized personnel under the control of inspectors from the Veterinary Service of the Local Sanitary Service (ASL 16-Padua), who are the local officers from the Ministry of Health. The use of the animals and the experimental protocol followed were approved by the ethical committee and by the animal welfare coordinator of the OPBA from the University of Padua. All procedures are specified in the projects approved by the Italian Ministero Salute, Ufficio VI (authorization number: 359/2015 PR; 81/2017 PR), and were conducted in accordance with National laws and policies (D.L. n. 26, March 14, 2014), and with the guidelines established by the European Community Council Directive (2010/63/EU) for the care and use of animals for scientific purposes.

### 2.4. Neuronal Cultures

Primary cultures of rat cerebellar granule neurons (CGNs) and spinal cord motor neurons (SCMNs) were prepared as described in [43,44,45].

### 2.5. Immunofluorescence and Axon Length Quantification

Immediately after plating, neurons were exposed for 24 h (or 5 days in microfluidic devices) to the indicated concentrations of NUCC-390 in the culture medium. Low-density neuronal cultures were used to test the effect of NUCC-390 on axon growth. At the indicated time points, neurons were fixed for 10 min with 4% (wt/vol) paraformaldehyde (PFA) in PBS, quenched (glycine 50 mM in PBS), and permeabilized with 0.3% Triton X-100 in PBS for 5 min at RT. After saturation with 3% goat serum in PBS for 1 h, samples were incubated with primary antibodies against β_3_-tubulin (1:200) in 3% goat serum in PBS overnight at 4 °C, washed, and then incubated with the corresponding secondary antibody Alexa-conjugated for 1 h at RT (1:200). Coverslips were mounted using Fluorescent Mounting Medium (Dako Agilent, Santa Clara, CA, USA) and examined by epifluorescence (Leica DMIRE2, Leica Microsystems, Wetzlar, Germany) microscopy. Th axonal length, evaluated by _3_-tubulin staining, was quantified with the ImageJ plugin NeuronJ following a semiautomatic tracing technique described in [46].

### 2.6. Evoked Junctional Potentials Recordings

Six to eight-week-old CD1 mice were anesthetized with isoflurane (Piramal Healthcare, Morpeth, UK), and locally injected in the hind limb with α-LTx (5 μg/kg in 15 µl of 0.9% NaCl, 0.2% gelatin solution). Four groups of mice (4 mice/group) were employed: group 1 received daily hind limb injections of NUCC-390 (3.2 mg/kg), group 2 was i.p. injected twice daily with AMD3100 (4 mg/kg), group 3 received a combination of the 2 treatments, group 4 only vehicle. Seventy-two hours later mice were sacrificed, soleus muscles dissected, immediately placed in an oxygenated (95% O_2_ and 5% CO_2_) experimental chamber, and subjected to electrophysiological measurements as described in [47]. Intracellular recordings of evoked junctional potentials recordings (EJPs) were performed at RT (20–22 °C) in oxygenated Ringer solution in current–clamp mode, adjusting the resting membrane potential with appropriate current injection to 70 mV. EJPs were elicited by supramaximal soleus nerve stimulation at 0.5 Hz using a suction microelectrode connected to an S88 stimulator (S88 Grass, Astro Nova, West Warwick, RI, USA) through a stimulus isolation unit (SIU5, Grass, USA). To prevent fiber contraction muscles were incubated for 10 min with 1 μM μ-Conotoxin GIIIB. Intracellular signals were amplified with an intracellular amplifier (BA-01X, NPI, Tamm, Germany), and fed to a PC for recording and offline analyses, using an A/D interface (NI PCI-6221,2.4). Changes in EJPs amplitude, indicative of differences in synaptic function and structure, were offline measured from recorded traces with Clampfit software (Pclamp9, Axon, San Jose, CA, USA).

### 2.7. NMJ Immunofluorescence

After electrophysiological recordings, soleus muscles were immediately fixed in 4% PFA (wt/vol) in PBS for 10 min at RT. After quenching in 50 mM NH_4_Cl in PBS, muscles were separated in bundles of 15 to 20 myofibers under a dissection microscope. Fiber bundles were permeabilized and saturated for 2 h in blocking solution (15% vol/vol goat serum, 2% wt/vol BSA, 0.25% wt/vol gelatin, 0.2% wt/vol glycine in PBS) containing 0.5% Triton X-100. Incubation with the primary antibody anti-syntaxin 1A/1B (1:200) was carried out for 48 h in blocking solution. Then samples were extensively washed and incubated with appropriate secondary antibodies (Alexa-488 conjugated, 1:200 in blocking solution), and with α-BTx Alexa-555 to stain post-synaptic nicotinic acetylcholine receptors (nAChR). Images were collected with a Leica SP5 confocal microscope (Leica Microsystems, Wetzlar, Germany), equipped with 40X HCX PL APO NA 1.4 objective. Power intensity was adjusted to avoid bleaching; laser excitation line and emission range were chosen according to each fluorophore in different samples to minimize bleed-through.

### 2.8. Statistics

The sample size was determined by analysis based on data collected by our laboratory in published studies. We used 4 mice/group for electrophysiological analysis. For neuron cultures studies, 2 replicates/experiment and 3 independent experiments were performed. Axon length quantification was conducted by an observer who was blind to the experimental groups. Data are reported as mean ± SEM. GraphPad Prism software was used for all statistical analyses. Statistical significance was evaluated using analysis of variance (ANOVA) with Tukey post-test or two-tailed, unpaired Student’s *t*-test depending on group number. Data were considered statistically different when *p < 0.05, **p < 0.01, ***p < 0.001, ****p < 0.0001.

## 3. Results

### 3.1. A Novel Synthesis of NUCC-390

**Figure 1:**
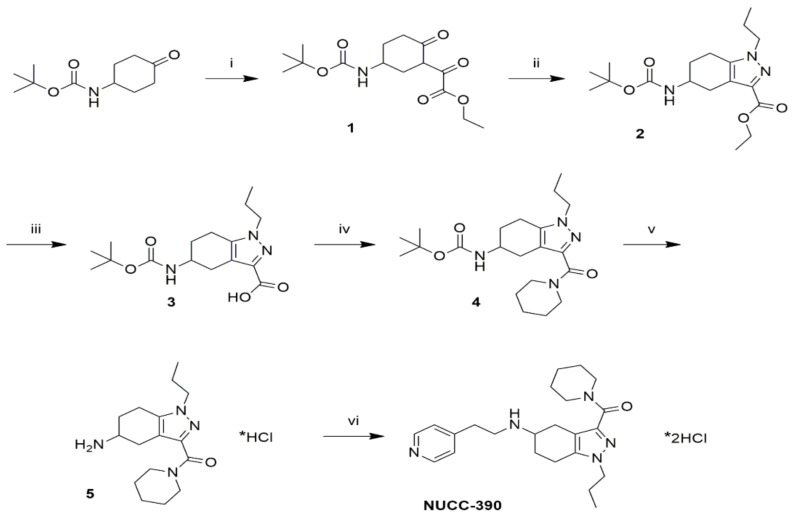
Scheme of the chemical synthesis of NUCC-390. Reagents and conditions: i) step a: LiHMDS, Et2O, and THF, −78 °C, 1 h; step b: diethyl oxalate, Et2O, −78 °C, 1 h; step c: RT, 3 h, 81% yield; ii) propylhydrazine *2HCl, K2CO3, EtOH, RT, o.n., 84% yield; iii) KOH aq, THF, MeOH, RT, o.n., 99% yield; iv) step a: DIPEA, HATU, DMF, RT, 15 min; step b: piperidine, RT, 45 min, 86% yield v) HCl 4 M in dioxane, DCM, RT, 3 h, quantitative yield; vi) step a: 4-vinylpyridine, acetic acid, MeOH, 80 °C, o.n.; step b: HCl 4 M in dioxane, 45% yield.

The outline of the chemical synthesis of NUCC-390 is shown in Figure 1. This procedure delivers the drug in high yields. It begins with 4-(Boc-amino)cyclohexanone to build the corresponding substituted oxo-(2-oxo-cyclohexyl)-acetic acid ethyl ester compound (1) through a Claisen condensation with diethyl oxalate. In the second step, the pyrazole ring was generated by condensation of the 1,3-diketone group of 1 with propylhydrazine to obtain (2). Following this step, the hydrolysis of the ethyl ester group of (2) in basic conditions led to the corresponding carboxylic acid compound (3), which was then coupled with piperidine to obtain the piperidine amide (4). Finally, cleavage of the tert-butyloxycarbonyl (Boc) group was performed to obtain (5), which was conjugated with 4-vinylpyridine through Michael addition to give NUCC-390.

### 3.2. NUCC-390 Induces Axonal Growth in Primary Cultured Neurons Via CXCR4

**Figure 2:**
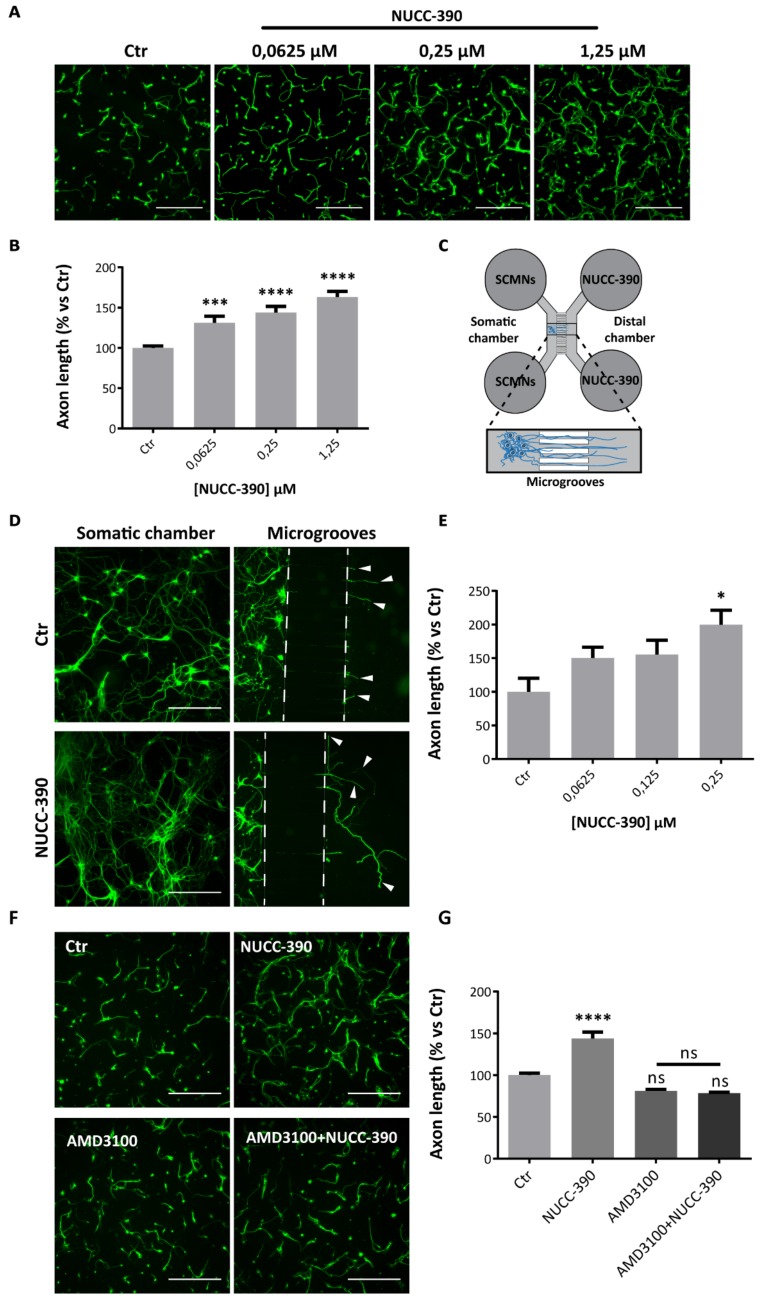
NUCC-390 stimulates axonal growth via CXCR4. (**A**) Cerebellar granule neurons (CGNs) were treated for 24 h with vehicle or NUCC-390 at the indicated concentrations, then fixed and imaged by β_3_-tubulin staining (green). Scale bars: 200 µm. (**B**) Quantitation of average lengths expressed as a percentage of vehicle-treated neurons (Ctr). Bars represent mean ± SEM from 3 independent experiments. ***p < 0.001, ****p < 0.0001. (**C**) Scheme of microfluidic devices used in the study. Spinal cord motor neurons (SCMNs) (blue ovals) plated in the somatic chambers (left) extend their axons through microgrooves toward distal chambers where NUCC-390 was added. (**D**) Representative pictures of SCMNs cultured in microfluidic devices and treated with vehicle (upper panels) or with 0.25 µM NUCC-390 (bottom panels) for 5 days. Left panels show the somatic chamber with SCMN cell bodies, right panels show the grooves across the two chambers, through which axons elongate to reach the distal compartments. Arrowheads point to the tips of the axons that have entered the distal compartment. Scale bars: 200 µm. (**E**) Quantitation of axon length upon 5 days treatment with different concentrations of NUCC-390 measured from the groove exit-point. Average values are expressed as a percentage of vehicle-treated neurons (Ctr). Bars are mean ± SEM values from 3 independent experiments. *p < 0.05. (**F**) Immediately after plating CGNs were treated either with vehicle (top left panel), or NUCC-390 (0.25 µM, top right), or AMD3100 (10 µM, bottom left), or their combination (bottom right). Twenty-four hours later, neurons were fixed and imaged for β_3_-tubulin (g*reen*). Representative pictures are shown. Scale bars: 200 µm. (**G**) Relative quantification. **** *p* < 0.0001; ns = not significant.

Recently, we showed that CXCL12α promotes the axonal growth of primary motor neurons in culture by interacting with the CXCR4 receptor [8]. We used the same experimental setting to test whether NUCC-390 acts similarly to CXCL12α, and monitored axon elongation as a readout of its biological activity through CXCR4. Figure 2A shows that the drug boosts axonal growth in cultured cerebellar granule neurons (CGNs). We chose these cells because they consist of >95% neurons, thus allowing one to exclude that the observed effect is indirect and mediated by other cells of the culture. NUCC-390 action is dose-dependent, and it reaches a plateau in the low µMolar range (Figure 2B). No toxicity of the drug was detected at higher doses (not shown). Noteworthy, the extent of maximum stimulation of axonal growth (24 h treatment) by NUCC-390 almost overlaps that of the recombinant chemokine (NUCC-390: 163% ± 6.9, *p* < 0.0001 vs. Ctr, *N* = 6; CXCL12α: 161% ± 2.6, *p* < 0.0001 vs. Ctr, *N* = 6).

A similar axonal growth-promoting effect was exerted by NUCC-390 in primary cultures of rat spinal cord motor neurons (SCMNs) tested under conditions close to the in vivo ones. Figure 2C shows SCMNs plated in the proximal chambers (somatic chambers) of a microfluidic device, separated from distal compartments by grooves of about 1 μm. This in vitro model mimics some aspects of motor neuron development and allows a direct and quantitative estimation of motor axon growth rate [48]. Two hours after plating the drug was added to the distal chamber, and neurons were allowed to grow for 5 days. The staining of the microtubule protein β_3_-tubulin of neuronal projections (panel D of Figure 2) shows that control neurons barely exit from the microgrooves to reach the distal compartment; on the contrary, NUCC-390-treated neurons develop very long and elaborated axons (arrowheads) that extend very far, clearly suggesting that NUCC-390 promotes axonal growth and elongation. In addition, in this system, NUCC-390 activity is dose-dependent, and it occurs in the same concentration range used for CGNs, as shown by the quantification reported in Figure 2E. In this respect, there appears to be no difference among motor neurons and brain neurons.

To ascertain that this axonal growth-promoting activity is indeed mediated by CXCR4, we performed a competition experiment between NUCC-390 and the high-affinity CXCR4 inhibitor AMD3100 [49]. The NUCC-390 effect on axon elongation is completely abolished by AMD3100 treatment, indicating that NUCC-390 stimulates axon growth via the specific engagement of CXCR4 (Figure 2F–G). Taken together these results document that NUCC-390 recapitulates the neurobiological activity of CXCL12α [8] and that, by interacting with CXCR4, it acts as an axonal growth factor for both central and peripheral neurons in vitro.

### 3.3. NUCC-390 Strongly Stimulates the Recovery of NMJ Function after Motor Axon Terminal Degeneration

**Figure 3:**
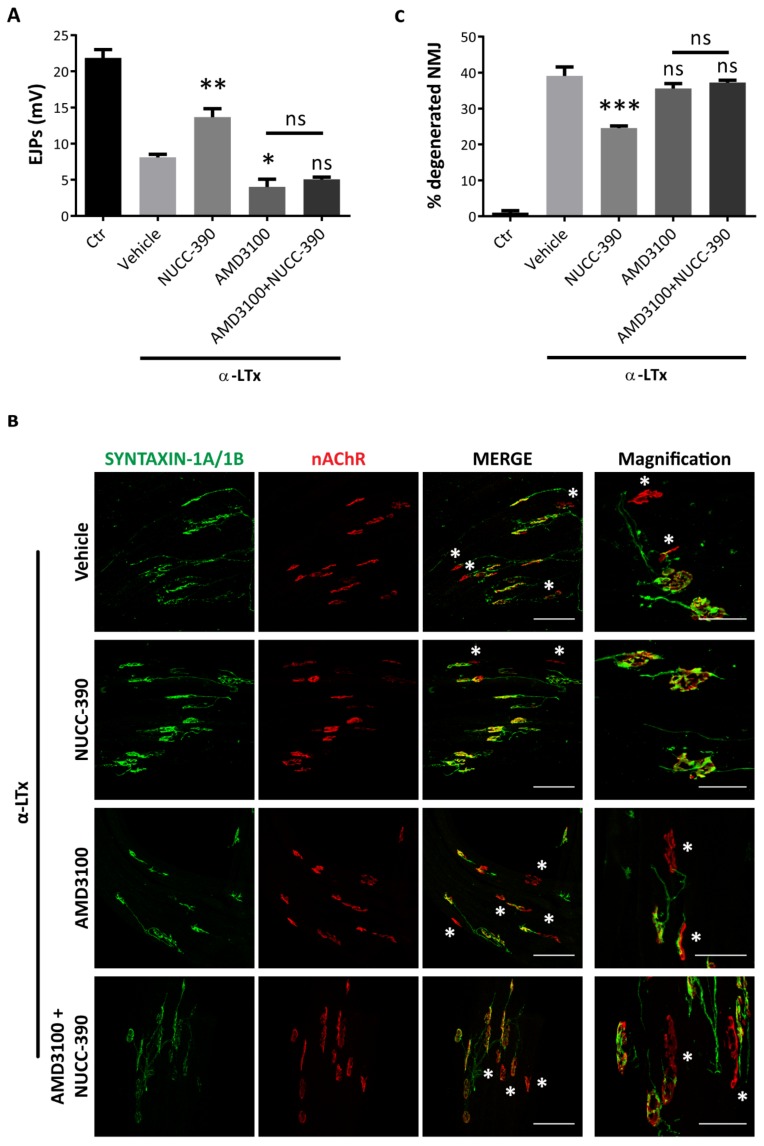
NUCC-390 promotes functional and anatomical recovery of the NMJ. (**A**) Mice locally injected in the left hind limb with α-LTx were daily treated either with vehicle, or NUCC-390, or AMD3100, or their combinations. Seventy-two hours later, soleus muscles were dissected, and EJPs recorded. *p < 0.05, **p < 0.01; ns = not significant. Bars represent mean ± SEM from 4 mice, 15 fibers analyzed/muscle. (**B**) The same muscles in A were paraformaldehyde (PFA)-fixed, and the neuromuscular junction (NMJ) stained for the presynaptic marker syntaxin-1A/1B (green), and for acetylcholine receptors (nAChR) by fluorescent α-BTx (red). Scale bars: 100 µm. Yellow signals in merge panels correspond to regenerated NMJ. White asterisks indicate still degenerated NMJ. Panels on the right display representative fields at higher magnification. Scale bars: 50 µm. (**C**) Quantitative analysis of degenerated NMJ in soleus muscles after α-LTx-induced injury ± treatments reported as a percentage of vehicle-treated mice. Bars represent mean ± SEM from 4 animals. ***p < 0.001. ns = not significant.

As CXCL12α stimulates the recovery of motor axon terminal function after degeneration induced by α-LTx [8], we tested whether NUCC-390 exerts a similar pro-regenerative activity in the same model. α-LTx is a pore-forming toxin that induces a fast, synchronous and complete degeneration of motor axon terminals, causing neuromuscular paralysis. Yet, in a few days in mice, degeneration is followed by a spontaneous recovery, which can be accurately and quantitatively monitored by recording the evoked junction potentials (EJPs) [50,51]. The analysis was performed 72 h after toxin injection, a time point where the functional activity of injured nerve terminals is about 50% rescued, thus allowing a better estimation of the efficacy of pro-regenerative treatments. Quantitative measurement of EJPs reported in Figure 3A shows that NUCC-390 strongly stimulates the recovery of neurotransmission after complete motor axon terminal degeneration, similar to what was previously reported for the recombinant chemokine [8]. Importantly, this pro-regenerative action was completely abrogated when mice were pretreated with AMD3100, a specific CXCR4 inhibitor. Noticeably AMD3100, by itself, lowers the physiologic regeneration process by preventing the activity of endogenous CXCL12α [8]. Imaging of the same muscles with specific antibodies against pre- and post-synaptic markers (Figure 3B), and the relative semiquantitative analysis (Figure 3C) confirm the electrophysiological results, showing a higher percentage of re-innervated NMJ in NUCC-390 treated muscles with respect to untreated samples. These data indicate that NUCC-390 action at the damaged NMJ closely resembles that of CXCL12α: by promoting recovery of function and structure of degenerated NMJ in mice, it represents a strong candidate to support peripheral regeneration also in humans.

## 4. Discussion

The main result reported here is that the small molecule NUCC-390 promotes the recovery of the physiological function of the murine NMJ after complete degeneration of its motor axon terminal. This action is CXCR4-mediated, as it is completely prevented by the selective CXCR4 antagonist AMD3100. In this respect, NUCC-390 acts very similarly to the natural agonist ligand, the chemokine CXCL12α. These results indicate that NUCC-390 acts in an opposite way of AMD3100, the bicyclam antagonist of CXCR4 and HIV entry inhibitor, which has been approved for clinical use with the names Plerixafor/Mozobil [52,53].

NUCC-390 is a small and water-soluble molecule, and it is expected to display better pharmacokinetics than the chemokine. In fact, CXCL12α is structurally complex (93 amino acids, 5 cysteins, 2 disulfide bridges), and it is very labile in body fluids resulting in a short half-life, owing to rapid post-translational modifications and hydrolysis that affect its activity [54,55]. These features strongly limit a potential pharmacological use of CXCL12α. Therefore, chemical compounds, such as NUCC-390, which recapitulate the pharmacological ability of CXCL12α to foster axonal regeneration, hold a great potential translational value for the treatment of peripheral nerve damages. Indeed several pathological conditions involve damage to peripheral neurons, from mechanical traumas to autoimmune diseases caused by antibodies acting on motor axon terminals, from neurotoxins to neurodegenerative diseases beginning from the axon terminal. All these pathologies are expected to benefit from this molecule that induces axon growth. As well, NUCC-390 may be beneficial for CNS pathologies implying axonal degeneration, given the axon growth-promoting activity that we observed on cerebellar neurons.

In the model of neurodegeneration used here, the axon terminal is induced to degenerate by a presynaptic neurotoxin which forms calcium conducting channels in the presynaptic membrane, causing cytosolic and mitochondrial Ca^2+^ overload, with consequent activation of various Ca^2+^ hydrolases and mitochondrial dysfunction [50,51,56,57]. This mechanism is common to several other NMJ pathologies, such as autoimmune neuropathies, including Guillain–Barrè and Miller–Fisher syndromes, where autoantibodies binding to presynaptic NMJ antigens activate the complement pore formation [58,59,60,61,62]. The same mechanism is also involved in the pathological action of snake neurotoxins acting presynaptically, that cause a reversible neuroparalysis in humans [63]. In addition, the initial stages of many neuromuscular–neurodegenerative diseases, such as amyotrophic lateral sclerosis and spinal bulbar muscle atrophy, are characterized by NMJ structural–functional instability, which then progresses in a dying back manner to motor neuron loss [64]. Although the biochemical mechanisms underlying these pathological conditions are largely unknown, drugs, such as NUCC-390, that stimulate endogenous receptors may be very useful in promoting nerve terminal stability and delaying disease progression.

## Supplementary Materials

The following is available online at https://www.mdpi.com/2073-4409/8/10/1183/s1. Supplementary File S1: Synthesis and characterization of piperidin-1-yl(1-propyl-5-((2-(pyridin-4-yl)ethyl)amino)-4,5,6,7-tetrahydro-1H-indazol-3-yl)methanone (NUCC-390).

## Abbreviations

CGNs, cerebellar granule neurons; EJPs, evoked junctional potentials; α-LTx, α-Latrotoxin; MAT, motor axon terminal; NMJ, neuromuscular junction; PNS, peripheral nervous system; PSCs, perisynaptic Schwann cells; SCMNs, spinal cord motor neurons.
