# Supplementary material for: An Agonist of the CXCR4 Receptor Strongly Promotes Regeneration of Degenerated Motor Axon Terminals
Source: Cells. 2019 Sep 30;8(10):1183. doi: 10.3390/cells8101183 (PMC6829515; doi:10.3390/cells8101183)
Supplement: Supplementary file 1 [file cells-08-01183-s001.pdf]

Supplementary material

Synthesis and characterization of piperidin-1-yl(1-propyl-5-((2-(pyridin-4-yl)ethyl)amino)-4,5,6,7-tetrahydro-1H-indazol-3-yl)methanone (NUCC-390)

Synthesis of ethyl 2-(5-((tert-butoxycarbonyl)amino)-2-oxocyclohexyl)-2-oxoacetate (**1**): A solution of *N*-4-Boc-aminocyclohexanone (1.0 g, 4.7 mmol) in THF (3 mL) and Et_2O (7 mL) was added dropwise to a stirred solution of LiHMDS (1 M in THF, 5.6 mL, 5.6 mmol) in Et_2O (4.4 mL) at -78 °C under N_2. The reaction mixture was maintained at -78 °C and stirred for 1 h. Diethyl oxalate (1.2 g, 8.2 mmol) in Et_2O (3 mL) was then added, and the solution was stirred at -78 °C for 1 h. The reaction mixture was then warmed to RT and stirred for further 3 h. The resulting solution was quenched with 1 M HCl (30 mL), diluted with a mixture of brine (40 mL) and H_2O (10 mL) and extracted with EtOAc (4 × 50 mL). The combined organic layers were washed with brine (100 mL), dried over anhydrous sodium sulfate, filtered and concentrated under reduced pressure. The residual yellow oil was purified by flash chromatography (eluted with DCM/EtOAc 92:8) to afford 1.19 g of **1** as a yellow oil (3.80 mmol, 81% yield). ^{1}H NMR (300 MHz, $CDCl_3$) δ 4.58 (d, J = 4.3 Hz, 1H), 4.32 (q, J = 7.1 Hz, 2H), 3.80 (s, 1H), 2.84 – 2.74 (m, 1H), 2.55 (dd, J = 7.4, 6.0 Hz, 2H), 2.34 (dd, J = 15.4, 8.7 Hz, 1H), 2.09 – 1.91 (m, 1H), 1.82 – 1.61 (m, 1H), 1.43 (s, 9H), 1.35 (t, J = 7.1 Hz, 3H); ^{13}C NMR (101 MHz, $CDCl_3$) δ 189.42, 180.33, 162.78, 155.20, 104.51, 79.64, 62.17, 45.59, 30.59, 29.96, 28.42, 26.96, 14.06. ESI-MS : m/z 314, $[M + H]^+$.

Synthesis of ethyl 5-((tert-butoxycarbonyl)amino)-1-propyl-4,5,6,7-tetrahydro-1H-indazole-3-carboxylate (**2**): Propyl hydrazine dihydrochloride (1.36 g, 7.84 mmol) and K_2CO_3 (2.31 g, 16.74 mmol) were added to a solution **1** (2.4 g, 7.6 mmol) in EtOH (67 mL) at RT under N_2 atmosphere and the resulting heterogeneous mixture was stirred overnight. The reaction mixture was concentrated to dryness, diluted with H_2O (30 mL) and extracted with EtOAc (4 × 30 mL). The combined organic layers were washed with brine (3 × 30 mL), dried over anhydrous sodium sulfate, filtered and concentrated under reduced pressure. The residual oil was purified by flash chromatography (eluted with petroleum ether/EtOAc 5:5) to afford 2.26 g of **2** as a pale yellow solid (6.42 mmol, 84% yield). ^{1}H NMR (300 MHz, $CDCl_3$) δ 4.57 (d, J = 5.8 Hz, 1H), 4.36 (q, J = 7.1 Hz, 2H), 3.99 (td, J = 7.2, 1.9 Hz, 2H), 3.93 (d, J = 7.1 Hz, 1H), 3.12 (dd, J = 16.4, 5.2 Hz, 1H), 2.66 (t, J = 6.4 Hz, 2H), 2.56 (dd, J = 16.4, 7.6 Hz, 1H), 2.20 – 1.95 (m, 1H), 1.94 – 1.71 (m, 3H), 1.44 (s, 9H), 1.36 (t, J = 7.1 Hz, 3H), 0.89 (t, J = 7.4 Hz, 3H); ^{13}C NMR (75 MHz, $CDCl_3$) δ 162.99, 155.43, 139.36, 138.63, 117.55, 79.54,

60.64, 51.44, 46.26, 28.73, 28.51, 28.19, 23.62, 19.31, 14.59, 11.20. ESI-MS : m/z 352, $[M + H]^+$.

Synthesis of 5-((tert-butoxycarbonyl)amino)-1-propyl-4,5,6,7-tetrahydro-1H-indazole-3-carboxylic acid (**3**): MeOH (10.5 mL) and an aqueous solution of potassium hydroxide 0.94 M (20.1 mL, 19.1 mmol) were added to a solution of **2** (2.24 g, 6.37 mmol) in THF (10.5 mL) and the resulting solution was stirred at RT overnight. The reaction mixture was then quenched with a solution of HCl 0.5 M (50 mL) and brine (50 mL) and extracted with EtOAc (4 × 100 mL). The combined organic layers were dried over anhydrous sodium sulfate, filtered and concentrated under reduced pressure to afford 2.05 g of **3** as a white solid (6.34 mmol, 99% yield). ^{1}H NMR (300 MHz, DMSO) δ 12.44 (s, 1H), 6.94 (d, *J* = 7.5 Hz, 1H), 3.94 (t, *J* = 7.1 Hz, 2H), 3.58 (s, 1H), 2.93 (dd, *J* = 16.2, 5.1 Hz, 1H), 2.83 – 2.69 (m, 1H), 2.67 – 2.54 (m, 1H), 2.38 (dd, *J* = 16.1, 9.2 Hz, 1H), 1.96 – 1.86 (m, 1H), 1.79 – 1.67 (m, 2H), 1.67 – 1.54 (m, 1H), 1.39 (s, 9H), 0.83 (t, *J* = 7.4 Hz, 3H); ^{13}C NMR (101 MHz, DMSO) δ 163.78, 155.06, 138.76, 138.63, 117.30, 77.59, 50.39, 46.28, 28.27, 28.11, 28.04, 22.75, 19.26, 10.95. ESI-MS : m/z 324, $[M + H]^+$.

Synthesis of tert-butyl (3-(piperidine-1-carbonyl)-1-propyl-4,5,6,7-tetrahydro-1H-indazol-5-yl)carbamate (**4**): HATU (0.87 g, 2.28 mmol) and *N*,*N*-diisopropylethylamine (DIPEA) (0.30 g, 2.28 mmol) were added to a solution of **3** (0.70 g, 2.17 mmol) in anhydrous DMF (10.5 mL) at r.t. and the mixture was stirred for 15 minutes. Piperidine (0.37 g, 4.35 mmol) was then added and the resulting solution was stirred for a further 45 minutes. The reaction mixture was quenched with a solution of 0.5 M HCl (50 mL) and brine (50 mL) and extracted with EtOAc (4 × 75 mL). The combined organic layers were washed with H_2O/Brine (1:1, 4 × 100 mL), dried over anhydrous sodium sulfate, filtered and concentrated under reduced pressure. The residue was purified by flash chromatography (eluted with chloroform/acetone 9:1) to afford 0.730 g of **4** as a white solid (1.86 mmol, 86% yield). ^{1}H NMR (400 MHz, $CDCl_3$) δ 4.59 (s, 1H), 3.90 (t, *J* = 7.2 Hz, 3H), 3.71 (d, *J* = 37.7 Hz, 4H), 2.97 (dd, *J* = 15.9, 4.9 Hz, 1H), 2.63 (t, *J* = 6.2 Hz, 2H), 2.51 (dd, *J* = 15.9, 7.7 Hz, 1H), 2.03 (s, 1H), 1.90 – 1.73 (m, 3H), 1.70 – 1.53 (m, 6H), 1.43 (s, 9H), 0.90 (t, *J* = 7.4 Hz, 3H); ^{13}C NMR (101 MHz, $CDCl_3$) δ 163.75, 155.50, 142.82, 137.65, 115.54, 79.43, 50.84, 48.27, 46.52, 43.37, 38.72, 28.54, 28.32, 26.93, 25.85, 24.89, 23.56, 19.34, 11.28. ESI-MS : m/z 391, $[M + H]^+$.

Synthesis of (5-amino-1-propyl-4,5,6,7-tetrahydro-1H-indazol-3-yl)(piperidin-1-yl)methanone (**5**): HCl 4M in dioxane (7.50 mL) was added dropwise to a solution of **4** (0.50 g, 1.28 mmol) in DCM (1.50 mL) under N_2. The resulting mixture was stirred for 3 h, then the solvent was evaporated under reduced pressure to yield 0.42 g of **5** (1.28 mmol, quantitative yield). This material was used for the next step without further purification. ^{1}H NMR (300 MHz, MeOD) δ 4.06 (t, J = 7.1 Hz, 2H), 3.82 – 3.69 (m, 4H), 3.64 – 3.48 (m, 1H), 3.09 (dd, J = 15.5, 5.2 Hz, 1H), 2.97 – 2.78 (m, 2H), 2.66 (dd, J = 15.6, 9.2 Hz, 1H), 2.37 – 2.22 (m, 1H), 2.09 – 1.92 (m, 1H), 1.91 – 1.78 (m, 2H), 1.76 – 1.54 (m, 6H), 0.93 (t, J = 7.4 Hz, 3H). 13C NMR (101 MHz, MeOD) δ 164.40, 142.26, 140.17, 114.76, 51.87, 48.45, 39.57, 27.42, 26.66, 25.41, 24.18, 19.98, 11.29. ESI-MS : m/z 291, $[M + H]^{+}$.

Synthesis of piperidin-1-yl(1-propyl-5-((2-(pyridin-4-yl)ethyl)amino)-4,5,6,7-tetrahydro-1H-indazol-3-yl)methanone (**NUCC-390**): A solution of **5** (0.31 g, 0.95 mmol), 4-vinylpyridine (0.20 g, 1.89 mmol), glacial acetic acid (0.06 g, 0.95 mmol) in 3.5 mL of dry methanol was stirred at 80 °C overnight. The reaction mixture was quenched with a saturated solution of $NaHCO_3$ (50 mL), diluted with brine (20 mL) and extracted with DCM (5 × 30 mL). The organic layers were collected and dried over anhydrous sodium sulfate, filtered and concentrated under reduced pressure. The residue was purified by flash chromatography (elution with chloroform/MeOH 9:1) to afford 0.17 g of **NUCC-390** free base (0.43 mmol, yield 45%). Then, to a solution of **NUCC-390** in dioxane (5 mL), HCl 4 M in dioxane (0.5 mL, 10 eq) was added dropwise, and the risulting mixture was stirred at r.t. for 15 minutes. The solvent was evaporated under reduced pressure and the resulting solid was lyophilized to afford the dichlorohydrate salt of **NUCC-390**. ^{1}H NMR (300 MHz, MeOD) δ 8.86 (d, J = 6.2 Hz, 2H), 8.17 (d, J = 6.3 Hz, 2H), 4.04 (t, J = 7.0 Hz, 2H), 3.74 (s, 4H), 3.65 – 3.57 (m, 3H), 3.48 (dd, J = 8.5, 4.8 Hz, 2H), 3.23 (dd, J = 15.0, 4.3 Hz, 1H), 3.05 – 2.83 (m, 2H), 2.77 (dd, J = 15.3, 9.4 Hz, 1H), 2.47 (d, J = 12.0 Hz, 1H), 2.17 – 1.98 (m, 1H), 1.92 – 1.77 (m, 2H), 1.76 – 1.54 (m, 6H), 0.92 (t, J = 7.4 Hz, 3H). ^{13}C NMR (101 MHz, MeOD) δ 164.60, 160.17, 142.65, 142.56, 140.01, 129.22, 114.50, 69.06, 56.07, 51.87, 45.42, 33.31, 27.41, 26.31, 25.44, 25.25, 24.23, 20.24, 11.32. ESI-MS : m/z 396, $[M + H]^{+}$.
